# Supplementary material for: List-wise learning to rank biomedical question-answer pairs with deep ranking recursive autoencoders
Source: PLoS One. 2020 Nov 9;15(11):e0242061. doi: 10.1371/journal.pone.0242061 (PMC7652278; doi:10.1371/journal.pone.0242061)
Supplement: Attachment — Submitted filename: Response to Reviewers.pdf [file pone.0242061.s002.pdf]

Dear Editor,

This is the response of our previous paper (PONE-D-19-28201) "List-wise Learning to Rank Biomedical Question-Answer Pairs with Deep Ranking Recursive Autoencoders”.

We give special thanks to the AE and the reviewers for their critical reading of our paper, appreciation, and suggestions to improve it.

After carefully considering the comments from the AE and the reviewers, we have made substantial modifications, especially for datasets and quantitative measures, and results analysis with intensive experiments. We list our major revisions as follows.

Major Modifications

(1) In the “Preprocessing and Pretraining” section, we have added more details about document retrieval，mainly related to query sequence processing.

(2) In the “Recursive Autoencoders and Variants” section, we have added a more visual description of Recursive Autoencoders so that readers can understand the following paragraph easily.

(3) In the “Semi-Supervised Modified RNNs for Ranking” section, we have added an explanation of why and how softmax layer was used， describe more details and give the reader a clearer understanding of our proposed model.

(4) In the “Comparisons with classical IR models” section, we have added data and analysis for this BioASQ 16-18.

(5) In the “Significance Testing and Experimental Analysis”, we have added a comparative analysis of our model about the reason why our framework performs better than other methods in two ways.

(6) We open source our code on GitHub.

https://github.com/lixuf/RAE-Recursive-AutoEncoder-for-bioasq-taskB-phaseA-snippets-retrieve-

Point by Point Response to AE and Reviewers

1.1. Responses to AE:

Thanks to AE’s helpful comments and suggestions. We have addressed all the issues raised by the AE in our responses to reviewers.

1.2. Responses to Reviewer 1:

(1) *The results are not well supported. To really understand why your method*

outperforms all the others, I would need more than MAP scores, for example, questions that other method got wrong and your method got right. Other aspects such as inference time would also be interesting.

Response: Thanks for the great comments.
We have added more details of the model in the article and added several analysis in the subsection “Significance Testing and Experimental Analysis” in “Results” section. The latter half of the subsection contains our analysis of the reason why our framework performs better than other methods in two ways.
This is the part we added and just mentioned: There is a typical model that gets result by measuring the similarities of term distributions [53]. It is difficult to consider comprehensively, although this paper proposed six aspects to measure the similarity between questions and answers. The model proposed by this paper also requires a lot of expertise and experimentation to determine which aspects to be used. Our proposed model encodes Q-S pair automatically instead of the similarities of term distributions. Our approach not only needs less expertise and experiments but also automatically selects the required information by neural network and has better result than his model. Another paper [26] also uses the similarities of term distributions, but they propose a similarity matrix generated by part-of-speech and similarity. The form of matrix can increase computing speed and their model performs better than ours. The reason why they get a better result mainly because they have a document retrieval model that can provide more accurate related documents than search engines, which is one of our weaknesses. Our other disadvantage is that we don't take full advantage of part of speech, position and similarity, but they do. Two methods provided us with improved ideas above. Combining our method with their method may achieve better results but requires more computing power and manpower.

(2) *The discussion does not go in-depth about what makes your method "more suitable than other vector representation models to a large extent" or that it "retains as much of the local semantics as possible".*

Response: Thanks for the great comments.
We have added some analysis and figure for your question in the subsection “Comparisons with variants of our approach” in “Results” section. We appreciate your comments for making our paper more convincing and detailed, and have carefully modified some details of the article.
This is the figure we added and just mentioned:

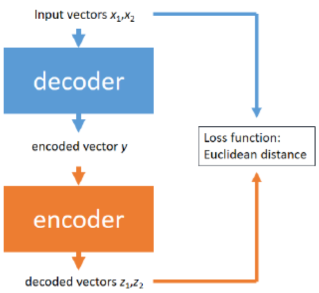

Fig 4. Input vector x_1, x_2 is the children mentioned in Recursive Autoencoders and Variants section, and encoded vector y is the parent mentioned in Recursive Autoencoders and Variants section.

This is the analysis we added and just mentioned: The loss function of RAE is the Euclidean distance between the input vectors and the decoded vectors, is shown in Fig 4. Therefore, the goal of each iteration of RAE is to obtain the decoded vectors with the highest similarity to the input vectors, so as to obtain the hidden layer state (encoded vector) that can be put into the decoder and get the vectors with a high degree of similarity to the input vectors. We can visually think of the encoder as a compression tool, which compresses the input vectors into a vector. It is worth noting that the structure of the encoder and the decoder must be consistent but the data flow should be opposite. This is why we can use the similarity between the decoded vectors and the input vectors as a criterion. Now, we were surprised to find that the input vectors x_1,x_2 encoder to vector y after a series of compute and the vector y decoder to the vectors that are highly similar to the input vectors x_1,x_2 again after a series of compute. The reason why the vector y can returned to the input vectors is the vector y has most of the features of the input vectors just like word embedding. So RAE can retain as much of the local semantics as possible, which is its goal also.

(3) *Why not show the results for BioASQ 16 -18? You also claim that your approach was evaluated on two other open domain tasks on the abstract, but this is never mentioned in the text.*

Response: Thanks for the great comments.

We supplemented the results for BioASQ 16-18 in the “**Comparisons with classical IR models” section**. About “*evaluated on two other open domain tasks on the abstract”*, we are very sorry, in fact we did not add these data to the paper. This is a mistake of our proofreading and we are very sorry for our rough proofreading. This abstract is from the previous version, which was not found and replaced during our proofreading. We are also actively conducting experiments in other domain, and have made some new findings, which we hope to describe in detail in the next paper. Sincerely, we are sorry again for such a mistake.

This is the BioASQ 16-18 data just mentioned:

Table 5. Comparisons with BioASQ 2016 participants.

System	Batch 1	Batch 2	Batch 3	Batch 4	Batch 5
our	**0.1247**	**0.1392**	**0.1701**	**0.2300**	**0.2401**
KNU-SG Team_Korea	0.1365	0.1590	0.1693	0.2305	0.2386
ustb_prir	0.0700	0.0884	0.1127	0.2298	0.2250
testtext	0.0641	0.0774	0.1069	0.1834	0.1694
WS4A	0.0039	0.0022	-	-	0.0144

Table 6. Comparisons with BioASQ 2017 participants.

System	Batch 1	Batch 2	Batch 3	Batch 4	Batch 5
our	**0.1402**	**0.1598**	**0.1524**	**0.1726**	**0.1847**
KNU-SG Team_Korea	0.1393	0.1734	0.1411	0.1385	-
ustb_prir	0.1620	0.2556	0.2452	0.2195	0.1851
testtext	0.1585	0.2523	0.2465	0.2043	0.1843
fdu	-	0.1664	0.1367	0.1534	0.1170

Table 7. Comparisons with BioASQ 2018 participants.

System	Batch 1	Batch 2	Batch 3	Batch 4	Batch 5
our	**0.1189**	**0.1628**	**0.1950**	**0.1102**	**0.0895**
MindLab	0.0004	0.2695	0.2113	0.1151	0.0874
ustb_prir	0.1193	0.1727	0.1953	0.1204	0.0798
testtext	0.1151	0.1463	0.2021	0.1213	0.0861
sdm	0.1089	0.1571	0.1533	0.0532	0.0441

(4) *Furthermore you also ignored other approaches to the snippet ranking task that have been proposed since the original tasks. For example this paper: xhttps://www.sciencedirect.com/science/article/pii/S1532046417300503 (Table 2) shows results for BioASQ 2015 snippet retrieval task superior to yours on every batch.*

Response: Thanks for the careful comments.

I'm sorry for the negligence of our previous research. We conducted an in-depth investigation based on the article you provided, and found an issue that we didn't realize before, and made our RELATED WORK section more perfect. Finally, thank you again. We have also added the corresponding paragraphs in RELATED WORK section.

This is the paragraphs we added and just mentioned: The third edition of the BioASQ challenge was hold in 2015. Sarrouti etc.[34] proposed using stemmed words and UMLS concepts as features for the BM25 model achieves good performances. The reason why they achieves good performances mainly they took full advantage of UMLS concepts and sentence components in both the document retrieval phase and the snippet retrieval phase. Their paper also proves that using the language resources of the sentence itself is sometimes as important as the model. A recent article also illustrates this fact [35].

(5) *Since the code is not provided, it would be difficult to test on other datasets or reproduce the results*

Response: Thanks for the careful comments.
We open source our code on GitHub.
https://github.com/lixuf/RAE-Recursive-AutoEncoder-for-bioasq-taskB-phaseA-snippets-retrieve-

(6) *Please make clear the distinction between Recurrent Neural networks and Recursive Neural Networks. You end up using RNN for both, while generally RNN is used for Recurrent Neural Networks.*

Response: Thanks for the careful comments.
We have corrected it and sorry for our mistake again.

(7) *There are occasional typos and language errors that should be revised, for example Line 250 "re relevant", while some expressions do not seem to be standard English: Line 147 "From our points".*

Response: Thanks for the careful comments.
Sorry for these missing words and expressions errors. We have revised our paper carefully, modified all related descriptions and very appreciated your careful observation.

1.3. Responses to Reviewer 2:

(1) *There is no error analysis documenting the reasons why the proposed system performs better*

Response: Thanks for the careful comments.
We have added more details of the model in the article and added several analysis in the newly written subsection “Significance Testing and Experimental Analysis” in “Results” section. The latter half of the subsection contains our analysis of the reason why our framework performs better than other methods in two ways.
This is the part we added and just mentioned: There is a typical model that gets result by measuring the similarities of term distributions [53]. It is difficult to consider comprehensively, although this paper proposed six aspects to measure the similarity between questions and answers. The model proposed by this paper also requires a lot of expertise and experimentation to determine which aspects to be used. Our proposed model encodes Q-S pair automatically instead of the similarities of term distributions. Our approach not only needs less expertise and experiments but also automatically selects the required information by neural network and has better result than his model. Another paper [26] also uses the similarities of term distributions, but they propose a similarity matrix generated by part-of-speech and similarity. The form of matrix can increase computing speed and their model performs better than ours. The reason why they get a better result mainly because they have a document retrieval model that can provide more

accurate related documents than search engines, which is one of our weaknesses. Our other disadvantage is that we don't take full advantage of part of speech, position and similarity, but they do. Two methods provided us with improved ideas above. Combining our method with their method may achieve better results but requires more computing power and manpower.

(2) *It is unclear if the code will be publicly available*

Response: Thanks for the great comments.
We open source our code on GitHub.
https://github.com/lixuf/RAE-Recursive-AutoEncoder-for-bioasq-taskB-phaseA-snippets-retrieve-

(3) *It is unclear how the system will perform outside the biomedical domain and on datasets other than the BioASQ task*

Response: Thanks for the great comments.
We are very sorry, in fact we did not add these data to the paper. This is a mistake of our proofreading and we are very sorry for our rough proofreading. This abstract is from the previous version, which was not found and replaced during our proofreading. We are also actively conducting experiments in other domain, and have made some new findings, which we hope to describe in detail in the next paper. Sincerely, we are sorry again for such a mistake.

(4) *The paper needs some proof reading for English*

Response: Thanks for the great comments.
Sorry that our expression is not very good, we have added more details in the new version and modified some expressions and grammatical errors. Thank you very much for your comments, and we will strengthen the proofreading of the article.

(5) *Why is a binary tree better than a parse tree for the RAE?*

Response: Thanks for the great comments.
A parse tree combination method is fixed, and binary tree combination method is determined by the neural network itself. It can combine vectors that are easier to combine and achieve the optimal substructure. We appreciate your comments for making us aware of the shortcomings of the part about the RAE of our article, and we have actively added instructions. Thanks again for your comments to make our article more detailed.
This is the part we added in "Recursive Autoencoders and Variants" **section：**
We chose a binary tree instead of a parse tree. Because the parse tree is built according to certain standards which and we can only encode vectors according to a fixed pattern, but the pattern of binary tree is selected by the neural network

itself. It can constantly encode more suitably and easier to encode vectors together.

1.4. Responses to Reviewer 3:

(1) *There is a regular typo along the article: many times, space character is wrongly added before commas or periods (even in the affliation part). line 250: re -> are*

Response: Thanks for the great comments.
Sorry for our mistake, we have revised our paper carefully and corrected the article. Thank you very much for your careful observation.
