# Supplementary material for: List-wise learning to rank biomedical question-answer pairs with deep ranking recursive autoencoders
Source: PLoS One. 2020 Nov 9;15(11):e0242061. doi: 10.1371/journal.pone.0242061 (PMC7652278; doi:10.1371/journal.pone.0242061)
Supplement: Attachment — Submitted filename: response to reviewers.pdf [file pone.0242061.s003.pdf]

Dear Editor,

This is the response of our previous paper (PONE-D-19-28201) "List-wise Learning to Rank Biomedical Question-Answer Pairs with Deep Ranking Recursive Autoencoders".

We give special thanks to the AE and the reviewers for their critical reading of our paper, appreciation, and suggestions to improve it.

After carefully considering the comments from the AE and the reviewers, we have made substantial modifications, especially for the result of Bioasq, examples analysis and proper comparison to other methods. We list our major revisions as follows.

1. **Major Modifications**

   (1) In the subsection "Comparisons with classical IR models" in "Results" section, we have added more details and analysis，mainly relating to the system performance and the "*extra resources".*

   (2) We have carefully revised the language and the result of Bioasq.

Point by Point Response to AE and Reviewers

1.1. Responses to AE:

Thanks to AE's helpful comments and suggestions. We have addressed all the issues raised by the AE in our responses to reviewers.

1.2. Responses to Reviewer 1:

(1) *examples/error analysis: unlike the teams that participated on the competition, it is possible to analyse the test set and give examples where the system performed well and where it failed. This is helpful to improve future approaches but it is still not present in this version.*

(2) *proper comparison to other methods: although the authors now show the results of their method on more recent editions of BioASQ; they justify the lower performance of the proposed method on the recent editions this way: "The decreases of improvements do not indicate the decline of robustness. The ultimate causes are the introduction of extra resources. Especially after BioASQ 2015, most of the systems based on extra resources contain a large amount of domain knowledge in biomedicine." this statement is vague and unfounded: what do the authors mean by "extra resources"? The description provided on the manuscript of the other team's systems mentions other resources used on the previous editions too: "Hana Database and BioPortal to retrieve biomedical concepts" and "look up the synonyms of the keywords in queries to select effective terms for query expansion". A more in-depth analysis is necessary to understand if the extra resources were really the cause of the improved performance of recent*

systems.

Response: Thank you and we understood that there should be more explanations.

For the first question regarding about the analysis, we have added more information about the system's performance and several analyses in the subsection "Comparisons with classical IR models" in "Results" section. The advantages and disadvantages of the model has be discussed to a further extend, as well as the aspects that are helpful to the model improvement.

For the second question about the method comparison, we have added more analysis to further explain the impact of extra resources on this task in the subsection "Comparisons with classical IR models" in "Results" section.

We truely appreciate your comments and question.

The following contents are added to the paper:

For example, introducing a pre-trained document retrieval model at the stage of retrieving documents can not only retrieve more comprehensively, but also reduce the probability of retrieving useless documents greatly. Using the more primitive search tool provided by pubmed, the top 100 relevant documents contain an average of 4.3 target documents, which can barely be called a comprehensive search. However, introducing too many useless documents brings a large error to the classification model. If extra resources of a pre-trained document retrieval model are introduced, the compression ratio of useful documents to useless documents can be reduced to 1:2 or even lower during the retrieval phase.

In addition, extra resources of language system, like UMLS, can help the model to better calculate the relationship between the problem and the paragraph through the connection between medical concepts or vocabulary. The word frequency is used to represent the degree of professionalism of the vocabulary, q-s pairs containing vocabulary with a word frequency less than 15 can be selected and retrieved in UMLS, and then be put the selected q-s pairs encoded with our model into the classification model containing Attention and output the results of the Attention. After standardizing the result of Attention, each word's the degree of influence in the q-s pairs of the final classification result can be obtained, which is a decimal between 0 to 1. A larger value indicates that the word has a greater influence on the final result. It is found that more than half of these highly specialized vocabularies have a low impact on the final result. But these specialized vocabulary and many professional concepts associated with the vocabulary are the key to a correct answer.

(3) *Furthermore although it is mentioned in the related work section, the results of the proposed method on BioASQ2015 are not compared with these: https://www.sciencedirect.com/science/article/pii/S1532046417300503.*

Response: Thanks for pointing this out.

Thank you for your excellent references. In the subsection "Comparisons with

classical IR models” in “Results” section and in “RELATED WORK” section, we compare the literature you provided with our method and thank you again for providing us with a good supplementary aspect of the paper.

The following contents are added to the paper:

In addition, extra resources of language system, like UMLS, can help the model to better calculate the relationship between the problem and the paragraph through the connection between medical concepts or vocabulary. The word frequency is used to represent the degree of professionalism of the vocabulary, q-s pairs containing vocabulary with a word frequency less than 15 can be selected and retrieved in UMLS, and then be put the selected q-s pairs encoded with our model into the classification model containing Attention and output the results of the Attention. After standardizing the result of Attention, each word’s the degree of influence in the q-s pairs of the final classification result can be obtained, which is a decimal between 0 to 1. A larger value indicates that the word has a greater influence on the final result. It is found that more than half of these highly specialized vocabularies have a low impact on the final result. But these specialized vocabulary and many professional concepts associated with the vocabulary are the key to a correct answer.

Sarrouti and El Alaoui proposed using stemmed words and UMLS concepts as features for the BM25 model, which achieved good performances. The reason why they achieved good performances mainly because that they made full use of UMLS concepts and sentence components in both the document retrieval phase and the snippet retrieval phase. Their paper also proved that using the language resources of the sentence itself was equally important as using the model.

(4) *English should be revised, particularly on the text that was added since the previous version.*

Response: Thank you for the comment.
We carefully went through the article and make many improvements. The sentences are now better, and the expressions are more appropriate.

(5) *Tables 5 6 and 7 - it is misleading to have the values of the first row bolded. I would suggest highlighting the highest value of each column instead.*

Response: Thanks for the careful comments.
We have made changes as you said, thank you for your comments.

1.3. Responses to Reviewer 4:

(1) *There are inconsistencies in the MAP scores of BioASQ participants reported in tables 2 to 7. A thorough check is needed to guarantee that the numbers are correct. 1.a) For example, the MAP scores in table 4 are significantly lower than the official results http://participants-area.bioasq.org/results/3b/phaseA/. This table*

even includes a score in the second batch for the oaqa system which didn't participate in that batch. In table 5, batch 5 the KNU team hast the score of the ustb team and vice versa. 1.b) It is not clear which teams are selected to be presented in the tables. For example in table 4 (batch 4 and 5) and 5 (batch 2,3,4, and 5) the winning fdu team (with the highest MAP score) is not included in the table. The same with the aueb team in table 7. 1.c) In addition, even for teams included in the tables, the reported MAP score is not always the best score achieved by the team. For example, in table 6, batch 1, the score 0.1620 is reported which corresponds to utsb_prir3, while utsb_prir2 achieves 0.1774.

Response: Thank you for the comment.
We carefully reviewed the paper and improved the content as much as we can. Once again thank you for the comments.

(2) *There are various expression and orthographic issues in the manuscript that need to be checked.*

Response: Thank you for letting us know this.
It is greatly appreciated that you helped us to reveal the errors and mistakes in the paper. We apologize for the limitation in the language, and the best effort were made to improve the paper. Thank you for reviewing the paper for us, and providing valuable suggestion and questions.
