# Supplementary material for: List-wise learning to rank biomedical question-answer pairs with deep ranking recursive autoencoders
Source: PLoS One. 2020 Nov 9;15(11):e0242061. doi: 10.1371/journal.pone.0242061 (PMC7652278; doi:10.1371/journal.pone.0242061)
Supplement: Attachment — Submitted filename: Response to reviewers.pdf [file pone.0242061.s004.pdf]

Dear Editor,

This is the response of our previous paper (PONE-D-19-28201) "List-wise Learning to Rank Biomedical Question-Answer Pairs with Deep Ranking Recursive Autoencoders”.

We give great thanks to the AE and the reviewers for their critical reading of our paper, appreciation, and suggestions to improve it.

After carefully considering the comments from the AE and the reviewers, we have made substantial modifications, especially for the content of table, and likewise the grammar. We list our major revisions as follows.

1. Major Modifications

(1) we have carefully proofread all of not only the content of tables but also the grammar in our paper.

Point by Point Response to AE and Reviewers

1.1. Responses to AE:

Thanks to AE’s helpful comments and suggestions. We have addressed all the issues raised by the AE in our responses to reviewers.

1.2. Responses to Reviewer 1:

Response: we extend the sincere appreciation for your reading.

1.3. Responses to Reviewer 4:

1) The main inconsistencies in the MAP scores of BioASQ participants presented in tables 2-7 have been removed. However, a) there is an error in table 6, batch 3 as the results reported are for a different task (document retrieval) instead of snippet retrieval, as done for the other batches and tables. b) The performance of the top participating systems is still missing in some tables/batches (In particular: table 3, batch 2, HPI-S1 MAP 0.048; table 4, batch 1, HPI-S2, MAP 0.0971; table 4, batch 3, oaqa, MAP 0.1969; table 4, batch 4, fdu2, MAP 0.2480; table 4, batch 5, fdu2, MAP 0.2424; table 5, batch 1, HPI-S2, MAP 0.1601).
In addition, a minor formatting error exists in Table 1, where the performance 0.1203 is bold instead of 0.1412 in BioASQ 2015, batch 5.

Response: Thank you for the comment sincerely.
We carefully reviewed the table and corrected all of the content carefully. We extend the deep apology for you as the careless errors we made.
.

2) There are still some syntactic issues in English that should be carefully re-checked.

Response: Thank you for your comment.
It is greatly appreciated that you helped us to reveal the errors and mistakes in the paper. We apologize for the limitation in the language, and the best effort were made to improve the paper . Thank you for reviewing the paper for us, and providing valuable suggestion and questions.
